# Addressing antimicrobial resistance in China: policy implementation in a complex context

**DOI:** 10.1186/s12992-016-0167-7

**Published:** 2016-06-06

**Authors:** Li Wang, Xiulan Zhang, Xiaoyun Liang, Gerald Bloom

**Affiliations:** School of Social Development and Public Policy, Beijing Normal University, Beijing, China; School of Health Management, Anhui Medical University, Anhui, China; STEPS Centre, Institute of Development Studies, University of Sussex, Brighton, BN1 9RE UK

**Keywords:** Antibiotics, Anti-microbial resistance, Regulation, Complex adaptive system, Incentive, Global public good

## Abstract

The effectiveness of antibiotics in treating bacterial infections is decreasing in China because of the widespread development of resistant organisms. Although China has enacted a number of regulations to address this problem, but the impact is very limited. This paper investigates the implementation of these regulations through the lens of complex adaptive systems (CAS). It presents the findings from reviews of relevant policy documents and published papers. The paper identifies different types of agent and explores their interaction with regard to the use of antibiotics and their responses to changes of the regulations. It focuses particularly on the impact of perverse financial incentives on overall patterns of use of antibiotics. Implications for the possibilities of nonlinear results, interactive relationships, and new pathways of policy implementation are discussed. The paper concludes that policy-makers need to better understand the objectives, incentives and potential adaptive behaviors of the agents when they implement interventions to improve antibiotic use and reduce the risk of emergence of resistant organisms.

## Background

China accounts for one-fifth of the world’s population and has made significant progress in improving public health since the establishment of the People’s of Republic of China (PRC) in 1949. Life expectancy has more than doubled from 35 years before 1949 to 74.8 years in 2010, and infant mortality has fallen from 200‰ before 1949 to 9.5‰ in 2013 [1]. The widespread use of antibiotics and the consequent reduction in rates of infectious disease has been an important contributor to this gain [2]. In recent years there has been growing evidence of bacterial infections that are resistant to the available antibiotics in many low-and-middle income countries (LMICs) [3]. This includes China, which is experiencing a growing problem with antimicrobial resistance [4, 5].

### High levels of antibiotic use

There is evidence of high levels of antibiotic use in China. A nationwide survey of 230,800 prescriptions in 784 community health institutions in 28 cities between 2007 and 2009, found that the number of antibiotic prescriptions was double that recommended by the WHO [6]. A study of prescriptions for outpatients and inpatients from 23 cities and 16 rural primary health care centers between 2009 and 2011 showed that 52.9 % of the outpatients were prescribed antibiotics, but only 39.4 % needed them on the basis of the clinical condition [7]. The same study found that 77.5 % of inpatients received antibiotics, but only 24.6 % were prescribed appropriately. Another study in two rural counties showed that 43 % of patients were prescribed antibiotics unnecessarily [8]. A recent study showed that combination use of antibiotics was found in 55.0 % of antibiotic prescriptions [7]. One study in 2008 found that 19 % of prescriptions for antibiotics were for two and 2 % for three or more [9]. A large proportion of antibiotics are administered by injection [7] including at village clinics [10].

### Antimicrobial resistance

Surveillance data show a growing problem with antimicrobial resistance. The China Bacterial Resistance Surveillance Study Group (BRSSG) found resistant strains of gram-positive cocci, including MRSA, ORSA, PRSP (R + I), and AREF during the early 2000s [11]. The National Antibacterial Resistance Surveillance Net (Mohnarin) was founded in 2005 by the Ministry of Health to monitor nationwide bacterial resistance. It began in 109 hospitals and has subsequently been extended to 1427 hospitals. Its 2004-05 Report showed that the prevalence of antibiotic resistance was much higher in China than in North America and Europe [5]. Subsequent reports have shown a growing trend in antibiotic resistance. The 2006-07 Report indicated that multiple-drug resistant bacteria, such as MRSA, ESBLs(+) enterobacteria, and non-fermenters, were common throughout the country [12]. The incidence of MRSA increased from 56.1 % in 2006-07 to 67.6 % in 2008, and the proportion of E. coli producing ESBLs went up from 35.3 % in 2006-07 to 56.0 % in 2008 and 70 % in the following years [13–15].

### Main antibiotic regulations and strategies

China has taken many measures to address antimicrobial resistance, often in response to global developments. It issued a Guideline for Antibacterial Use in Clinical Practice in 2004, in response to the 2001 WHO Global Strategy for the Containment of Antimicrobial Resistance [16]. In 2012 it enacted the Measures for the Management of the Clinical Application of Antibacterial Drugs soon after the WHO made “Antimicrobial resistance: no action today, no cure tomorrow” the key health issue of the 2011 World Health Day [17]. Table 1 summarizes measures that have been taken to address the problem of antimicrobial resistance.

**Table 1:** Main antibiotic regulations and strategies in China

• *Guidelines for antibiotic use in clinical practice*, enacted in 2004 as the first national guidelines for the clinical application of antibacterial drugs, categorizing antibiotics as non-restricted, restricted, and special use, NHFPC, SATCM, HDGLM	
• Measures to forbid retail pharmacies from selling antibiotics without prescriptions, 2004, CFDA	
• *Notice on establishing a surveillance net for the clinical application of antibacterial drugs and Mohnarin*, 2005, NHFPC, SATCM, HDGLM	
• *Notice on further strengthening the management of the clinical application of antibacterial drugs*, 2008, the General Office of NHFPC	
• *Notice on the management of the clinical application of antibacterial drugs*, 2009, the General Office of NHFPC	
• National Essential Medicines Policy (NEMP), 2009, the State Council of China. Advocating prioritizing the use of essential medicines and canceling the 15 % mark-up policy in all government-owned primary healthcare(PHC) facilities by the end of 2011.	
• Measures for the Management of the Clinical Application of Antibacterial Drugs, 2012, NHFPC	
• Three year national special campaign on the clinical application of antibacterial drugs, 2011-2013, NHFPC	
• National education program of “66 Items Related to Citizen’s Health Literacy in China”, viewed as a program that every citizen should know basic health information, 2008(revised in 2015), NHFPC	
• Health China Propaganda Program, 2013, NHDPC, CFDA and CAST	
Source: government documents	
NHFPC -the National Health and Family Planning Commission	
SATCM -the State Administration of Traditional Chinese Medicine	
HDGLM-the Health Department of General Logistic Minister	
CAST -the China Association for Science and Technology	
CFDA-the China Food and Drug Administration	
Mohnarin- Ministry of Health National Antimicrobial Resistance Surveillance Net	

These interventions have had some success, but antibiotics abuse is still common in China. Although, NHFPC surveillance data showed a fall in the percentages of antibiotics used for both inpatients and outpatients between 2010 and 2014, overuse of antibiotics and IV infusions was still a major problem [18]. Total drug expenditure from more than 770 surveillance sites nation-wide almost doubled between 2010 and 2013. Reynolds et al. [19] argue that the implementation of national guidelines on antibiotic use has been fragmented and incomplete, and China has made only limited progress in containing antibiotic resistance [19]. Li et al. [6] argue that oversight of antibiotics use is lax. A manager at the NHFPC indicated that China does not yet have a well-organized system to promote the rational use of antibiotics [18].

Many studies have attempted to understand the implementation of these regulations and strategies. Zhang et al. [20] investigated outpatient antibiotic use by pediatric hospitals and found that the guidelines have a positive impact on the use of narrow-spectrum antibiotics but not of broad-spectrum ones. Jin et al. [10] used a backward mapping methodology to explore users’ perceptions and their impacts on technology regulation. Li et al. [6] argued that removing financial incentives from prescribing, as well as more vigorous supervision of the pharmaceutical market and prescribing behavior were needed in order to curb overuse of antibiotics. Gu [21] argued that the underlying cause of over-prescribing was inappropriate government regulation of prices for health services and medicine. Liang et al. [22] studied the impact of government structure reform on antibiotic prescriptions in community health centers.

These publications bring different perspectives for analyzing the influence of regulation on antibiotic use. One way to bring these perspectives together is by viewing antibiotic use as a complex system [16, 23], in which many actors at different government levels and in a variety of institutions, including hospitals, pharmacies, manufacturers, insurance agents, and consumers, respond to interventions by adapting their behaviors in ways that have positive and negative effects [24]. Moreover, some actors function largely outside the health regulatory framework [25]. This paper applies the concept of a complex adaptive system (CAS) to analyze the implementation of regulations and strategies regarding antibiotic use.

## Antibiotic regulation in a complex system

One important element of a complex adaptive system is the existence of many interacting agents, capable of self-organization and responding to disruptions through interactive behaviors [26, 27, 28]. The interactions between agents are complex and dynamic so it is difficult to control or predict the result of a specific intervention [26, 27]. There are feedback loops and interdependencies between parts of the system, and there is often no direct cause and effect relationship [29, 30]. In a complex world, strategy is a series of processes for managing change [31].

The WHO defines a health system as ‘all organizations, people and actions whose primary intent is to promote, restore or maintain health’ [32]. There is an increasing recognition of the degree to which health systems exhibit the characteristics of a complex adaptive system [26, 33]. This can explain why an insignificant stimulus can sometimes create rapid change at scale and why at other times major interventions result in modest outcomes or unpredicted consequences [26, 30]. Health interventions, such as legislative and educational efforts, are inherently complicated to design and evaluate correctly and complex science can provide a useful framework for designing and implementing them [34, 35].

A growing body of literature applies the concept of CAS to health systems and their transformation. There have been publications on the design of primary care practice [30, 36–38], practical solutions in clinical care [39], designing evaluation programs for health interventions [34, 35, 39], explaining the relationship between management practice and health outcomes in nursing homes [40] and variation and change in family practices [41]. These studies frame the interacting parts and agents in the health system in different ways in analyzing how adaptive activities and interactive relationships influence the implementation of policy interventions.

Regulation of antibiotic use has to balance the need to ensure that people have access to appropriate antibiotics, when they need them, whilst preserving the efficacy of these products, as a global public good [42, 43]. Overuse of antibiotics results from a combination of inappropriate financial incentives, lack of surveillance information on resistance and the understandings of users and suppliers of these products, amongst other things [23]. The coordination of different professions and authorities, decentralized organizations with regional groups, and news media are important factors for successfully changing antibiotic use [44]. A systems approach can help with the identification of drivers and obstacles to effective interventions [23].

China’s health system is experiencing a number of continuous and rapid reforms. It is important to situate an effort to alter antibiotic use in this context. Several studies have contributed to our understanding of this context using the concept of CAS. Bloom et al. [45] explore health market regulation and suggest approaches proven to work well in one context may not be effective in other contexts. They argue that regulatory strategies need to be based on an understanding of local institutions. Xiao et al. [24] use the lens of CAS to evaluate the implementation of essential drug policies in Chinese rural areas. The authors found that divergent and unpredicted outcomes emerged because of the interaction between agents. Zhang et al. [46] apply CAS theory and resilience thinking to an analysis of the evolution of the system of rural health finance. They argue that policy development and implementation is the result of the responses of many interacting agents to a dynamic context and the attempts of government to respond to these adaptations.

This paper explores the adaptive behavior of different agents, interacting relationships, and the incentives likely to influence the implementation of policies to improve antibiotic use. It views providers and users of antibiotics as key actors in the system and explores the impact of their adaptation to new government regulations. It focuses on antibiotics for human use rather than for animal husbandry and aquaculture. This paper does not consider related issues, such as environmental spread of antibiotics and antibiotic-resistant bacteria, infection prevention and control, sanitation and hygiene, large-scale migration, bacteria culturing in laboratories, and rapid diagnosis [19].

## Methods

This paper explores the implementation of antibiotic regulations in China as a case study that identifies and analyzes factors that explain the limited impact on antibiotic use in a complex context. The data were collected from the following sources: (1) national policy documents about antibiotic use, and related polices in a broader area, (2) published studies in the scientific literature, (3) and other documents.

**Fig. 1:**
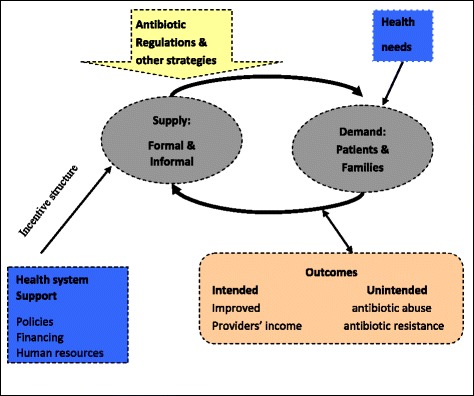
Conceptualising implementation of regulations and other strategies in CAS

Figure 1 presents a framework that can be used to analyze the implementation of antibiotic regulations. The key agents are involved in the relationship between supply and demand. The factors that support the health market can set a positive or negative incentive structure. Under the totality of an outside incentive structure and policy intervention, all types of agents will adapt themselves to the environment through interactive behavior and strive to protect their interests, which may be consistent or contradictory to the policy goal. Intended or unintended outcomes may appear.

## Findings and discussion

### Many agents in the system

Many types of agents, including governments, producers and distributors of pharmaceuticals, hospitals, patients, insurance agents and many informal actors are involved in the system of antibiotic use [45].

Complex and Fragmented Policy Provision. Government is responsible for making, implementing, and regulating policy. According to Fang [47], Chinese health care regulatory institutions include the system of public health administration, the auditing system, the price control regulations, the Food and Drug Administration (FDA), the Industry and Commerce Administration (ICA) system, and other informal regulatory arrangements. In the context of antibiotic use, the public health administration system is responsible for most of the direct regulations and strategies mentioned above; the FDA oversees organizations and individuals who address buying, selling, and prescribing drugs based on *Drug Management Law* and its Implementation Statute; the Price Control System establishes the prices of health services and drugs (at present, the government is exploring ending price regulation of most of medicines) and the ICA system has the duty to oversee private health facilities.

Many Providers and Multiple Channels to Users. All types of producers, distributors, health facilities, and retail pharmacies can be seen as antibiotic providers. There are six types of medicine circulation path from medicine providers to patients: (1) producers-distributors-health facilities-patients, (2) producers-distributors-patients, (3) producers- distributors-retailers-patients (4) producers-health facilities-patients, (5) producers-retailers-patients, (6) producers-patients [48]. Before 2009, most drugs were distributed from producers to wholesalers, then to hospitals or retail pharmacies, and finally to patients. Since the NEMP was implemented in the 2009 health system reform, individual health facilities have been forbidden from purchasing essential drugs directly from producers or wholesalers, but they are allowed to purchase through provincial public bidding [49]. By the end of 2015, there were 466,546 drug trading enterprises, including 13,508 wholesalers, 4,981 private retail chain enterprises, 204,895 chain drugstores and 243,162 standalone drugstores [50]. There were 985,000 healthcare facilities in 2014 [51], and their total expenditure accounted for 62.56 % of the National Total Expenditure on Health(TEH) in 2013 [52]. Public facilities have a dominant position, accounting for 86 % of hospital beds. Pharmacies owned by hospitals account for approximately 80 % of the drug market in terms of value [49]. Health facilities have a bilateral monopoly position in the Chinese medicine market, which means they have both a buyer-monopoly position because of powerful purchasing ability, and a seller-monopoly position because of dominating prescription rights [21]. Drug producers and distributors are not only the main suppliers of antibiotics to health facilities and retail pharmacies, they are also increasingly selling directly to patients through the internet. Retail pharmacies are becoming important supplier of antibiotics. The proportion of their drug sales in national total drug sales has a steadily rising trend, increasing to 30.14 % in 2012 [52]. Other semi-formal or informal providers, such as village clinics and barefoot doctors, and some illegal private clinics and pharmacies add complexity to the system.

Patients are key actors. Their understandings of the appropriate use of antibiotics strongly influence patterns of use [10]. Self-medication is common because it allows people to avoid the crowding and high costs of hospitals.

Insurance agents can also influence antibiotics use. The payment system can influence doctors’ behaviors. For example, Fee for service, in which providers are retrospectively reimbursed for each service rendered, can cause over-treatment.

### Confusing and complicating incentive mechanisms

The public health administration, including central and local health and family planning administration departments, is the main government arrangement for the regulation of health services. It also acts as the administrative leader and advocate of public healthcare facilities. This means that the health administration department needs to balance conflicting objectives between maintaining the operation and income of public health facilities and supervising their activities to meet people’s interests.

Since the beginning of the transition from a planned to a market economy, government subsidies to public health-care facilities have been reduced substantially, while the government has set the prices for health services in public facilities below the economic cost. Health facility managers have had to find other ways to meet their financial needs [21]. In 1992, the State Council proposed that healthcare facilities should “rely on government subsidies for capital investment, but rely on user charges and drug mark-ups for operational activities”. User charges and drug mark-ups became major sources of hospital finance [49]. Drug sales have become a very important source of revenue. These market-like activities have created strong incentives for selling high volumes of expensive drugs and diagnostic tests.

Until recently, secondary and tertiary general hospitals have been permitted to add a 15 % mark-up to the wholesale price of drugs, and in practice, the mark-up has been higher. This has created an incentive for health facilities to prescribe more medicines and also has encouraged the facilities to purchase drugs at relatively high prices and prescribe more expensive products, rather than the cheapest antibiotics. The producers and distributors of pharmaceuticals have sought profit by winning bids for government procurement. The 15 % mark-up policy meant they would do their best to persuade public hospitals to purchase expensive drugs and win the bid at a higher price, often offering kick-backs [21]. It is not surprising that many inexpensive, but effective, drugs have disappeared from the market because there was little incentive for drug producers and distributors to supply them. The National Development and Reform Commission (NDRC) has conducted specific types of medicine price-reduction activities more than 30 times since 1997, but they have had little long-term impact, because of the adaptation of agents to the altered incentives [21].

In recent years, primary healthcare facilities in China have been required to eliminate the 15 % mark-up, in order to cut the linkage between prescriptions and revenue generation, but the effect has not been straight forward. There have been a few studies of the effect of the National Essential Medicines Policy (NEMP) on the use of antibiotics in primary health care facilities. The effect has been moderate, although the results of these studies are inconsistent. Shan [53] and Yang [54] found that the NEMP had a positive effect on improving rational usage of antibiotics, although irrational usage still remained serious.. Jin et al. [55] indicated that the NEMP had significantly increased the joint usage of antibiotics, steroids and intravenous infusion in township health care, after evaluating 76,451 prescriptions based on a pre-post with non-equivalent control design and logistic regression. Song et al. [56] collected 28,651 prescriptions from 146 township health centers in a field survey conducted in four provinces of China in 2010-2011 and found little impact of the NEMP on the average number of antibiotics per prescription, but the percentage of prescriptions including antibiotics decreased a little (from 60.26 % to 58.48 %). This paper argues that moderate effect is due to the payment mechanism that was put by reimbursing facilities on the basis of average drug profits in the preceding three years.

The public health administrative agency has taken the lead in formulating most of the regulations and strategies contained in Table 1. As the sponsor of public health facilities, it has the incentive to ensure their status and the income of the healthcare workers. This may have limited their efforts to implement the regulations they formulated. Although the FDA and ICA systems are also involved in regulating drug use, neither are responsible for how medicines and drugs are prescribed and used at health facilities [47].

Medical insurance payment mechanisms have a different incentive structure. For a long time, fee-for-service has been the dominant way for reimbursing healthcare costs. This payment mechanism allows physicians to prescribe more treatments, and patients have no incentive to refuse excessive healthcare services. A number of reform efforts have been attempted to reduce its influence, such as moving towards bundled payments and capitation. Nonetheless, the effectiveness of these efforts has been limited because of the way they were implemented [57]. Hospitals cannot retain the savings, when there is a balance and they do not need to pay themselves when there is overspending [58].

**Fig. 2:**
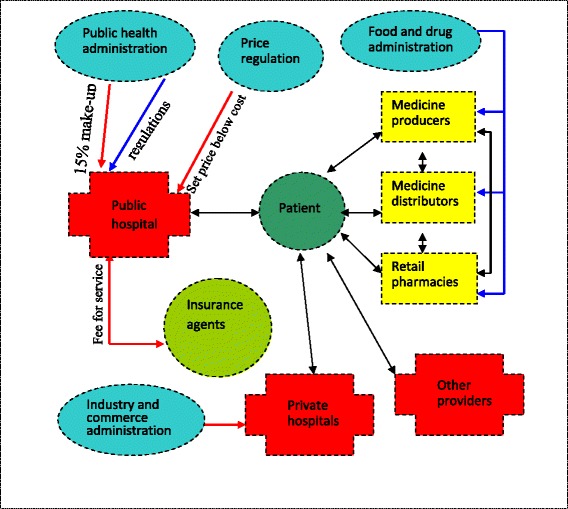
Different actors in complexity system and their linkages

Figure 2 presents the main actors and their linkages. A red arrow indicates a perverse incentive and a blue arrow means interventions, such as regulations and the black arrow indicates a supply and demand relationship.

### Interaction of agents and antibiotic use

Pressure on doctors by patients contributes to high levels of antibiotic use. Some people ask for sophisticated and expensive antibiotics or intravenous drips because they believe they are especially effective in curing infectious diseases [10]. People may prefer some specific drugs because of their experience. If a patient has recovered from sickness after taking a certain drug, he or she may ask the doctor to prescribe the same drug when they have similar symptoms again [10]. Moreover, doctors may be afraid of medical disputes and lawsuits, and might use preventive antibiotics [59], in contradiction to guidelines that state that antibiotics should not be used as prophylactics except during surgery. In fact, use is popular even if the patient just has a common cold [60]. With all these types of pressures combined with a perverse financial incentive system, doctors are more likely to over-prescribe antibiotics because it can ensure their income, prevent disputes and lawsuits, and satisfy the patients.

Retail pharmacies are becoming an important source of antibiotics. Most try to be an accredited supplier to beneficiaries of medical insurance. Patients tend to prefer retail pharmacies because they do not need to wait in line for a doctor’s prescription and wait again at the hospital pharmacy [58]. The average availability of medicines in retail pharmacies tends to be higher than in public hospitals [61]. In 2004, the government issued a regulation making antibiotics a prescription-only drug, however, retail pharmacies often do not ask clients to show a prescription [62]. Or, retail pharmacies can circumvent the regulation by employing a doctor to prescribe antibiotics, as required [60]. Several studies have found the percentage of antibiotics sold in retail pharmacies was high, and selling antibiotics without prescriptions was widespread [63–65]. Moreover, many retail pharmacies do not provide advice by professional pharmacists [66]. A study by Jiang [61] has shown that only 0.9 % of pharmacies could provide completely correct guidance regarding antibiotic use.

Online pharmacies are becoming an important source of antibiotics [67]. It is easy to buy antibiotics without a prescription from online pharmacies. Mechanisms to supervise online activities are not well-established and there is evidence of a growing problem with the supply of fake drugs through the internet [67, 68]. By June 2011, the FDA had closed 709 fake-drug making or selling sites, the total amount of which was approximately 0.7 billion RMB, and 70 % of the fake drugs were sold through the internet [69]. Regulating online drug supply is very much needed.

Patients’ ignorance may strengthen the above interaction between doctors and patients, or between drug stores and patients. In China, a large number of patients are ignorant about antibiotics. Many people have never heard of antibiotics but associate them with *xiaoyanyao,* which actually consists of antipyretic-analgesic and anti-inflammatory drugs, and steroid hormones. They are not concerned with antimicrobial resistance and believe new generations of antibiotics will be invented continuously [10]. A cross-sectional study found that most parents in rural areas had a low level of knowledge about antibiotics, and self-medication for children was common [70]. Even university students do not have adequate knowledge of antibiotics, and the proportion of self-medication is high [71]. Ignorance may lead patients to misunderstand “good drugs or quality services” as these can cure illness quickly. Common self-medication means a large number of people will pursue antibiotics through drug stores (online or not). Consequently, a strengthening behavior towards antibiotic abuse by both providers (hospitals or drug stores) and patients will be seen.

## Implications for the implementation of antibiotic regulations and other strategies

### Nonlinear results in the implementation process

Complexity can lead to unexpected consequences, which means there may not be a simple linear relationship between the effect and implementation of regulations. In theory, Government should make regulation policies, then administrators and regulators monitor and supervise their implementation. Meanwhile, health and medicine providers should supply antibiotics rationally according to these regulations. Finally, patients should accept the advice of these providers and use antibiotics according to the guidelines. The result should be the rational use of antibiotics. In practice, the implementation of regulations has resulted in a series of nonlinear outcomes, due to the adaptive activities of different agents, in protecting their interests. For example, most of the regulations are aimed at changing the behavior of doctors. However, the incentives the doctors face have led many of them to promote consumption of large volumes of expensive products. It will be difficult to change behavior, without paying attention to these incentives. The same applies to the rules that require pharmacies to ask for a prescription. Several national health education programs have been organized to improve people’s basic health knowledge, such as ‘66 Items Related to Citizen’s Health Literacy in China’ held in 2008 and revised in 2015 by NHDPC, and “Health China Propaganda Program” in 2013 hosted jointly by NHDPC, CFDA and CAST. Despite these efforts, most people still know little about antibiotics.

Policy makers and regulators should not think their job is done when policies and regulations are developed. Without taking into account the various agents’ adaptive behaviors and possible nonlinear results, to adjust the specific implementation plans, the planned results will not be achieved automatically.

### Understanding interactive relationships between types of agents

We identify two types of interactive relationship regarding antibiotic use and regulation: one is the role of health administration departments in both sponsoring and monitoring the performance of medical institutions; the other is the strong supply-demand relationship between providers and consumers of antibiotics. The first relationship could cause a lax implementation of regulations on antibiotic use. Health administration departments are under pressure to ensure the financial health of hospitals and also to oversee their prescription behavior. The second relationship could increase the difficulty of regulation. Information asymmetry is a nature of healthcare industry. This may be more serious in China because of the population’s low level of knowledge about antibiotics. Induced demand from providers is widespread, and it is very hard to supervise all sorts of over-prescription activities, because it is time-consuming and costly.

### Developing new pathways based on agent’s adaptive behavior

Policy implementation is always influenced by the adaptive responses of actors and their relationships [24]. For instance, under the pressure of the low-price-regulation of health services and the “15 % make-up policy”, doctors have a financial incentive to over-prescribe antibiotics and respond passively to regulations. The Government is now trying to implement a zero mark-up policy, but Xiao and her colleagues [24] found that hospitals have responded to this policy by creating new incentives to provide high levels of inpatient care. Jin et al. [55] found that doctors increased intravenous services to compensate revenue when drug income decreased due to the NEMP. It is necessary to anticipate possible range of behavioral responses in more complex context, and to take consideration on interests of all stakeholders.

## Conclusion

Antibiotic regulations and other strategies have been developed continuously in the last ten years, but high levels of antibiotic use have persisted and the problem of resistance has grown worse. The effectiveness of policy implementation is limited due to the complexity of China’s health system. Various agents with different objectives and in multiple interactive relationships are involved in policy implementation and have adapted to changes that affect their interests. Using complex adaptive system framework to better understand each type of agents’ interests, responses, and their adaptive behaviors could help us to better understand challenges and obstacles of coping with the issue of high levels of antibiotic use and antimicrobial resistance.

The paper maps out the agents, their incentives and behaviors from the perspective of complex adaptive system. Based on the above analysis, we have identified four key routes in changing the antibiotic use and antimicrobial resistance situation.

One route is to alter finance incentives in secondary and tertiary hospitals. In 2012, secondary hospitals treated 1.055 billion outpatients and tertiary hospitals treated 1.087 billion; 41.5 % and 42.7 % of the total, respectively [49].

The second route is to address adaptive behaviors in primary health facilities after the “zero mark-up” intervention. Changing the financing structure of the primary health facilities to reduce the strong incentives of selling drugs is essential.

The third route is to provide effective health education, especially to inform rural residents about antibiotics and antimicrobial resistance. And the last route is pay attention to the large number of informal suppliers of antibiotics outside the formal regulation system, especially through measures to regulate online suppliers of drugs.

## Abbreviations

BRSSG, Bacterial Resistance Surveillance Study Group; CAS, complex adaptive system; CMS, Cooperative Medical System; ICA, Industry and Commerce Administration; LMICs, low-and-middle income countries; Mohnarin, National Antibacterial Resistance Surveillance Net; NCMS, New Cooperative Medical System; NDRC, National Development and Reform Commission
